# Comparative Genomic Analysis of Multi-Drug Resistant *Pseudomonas aeruginosa* Sequence Type 235 Isolated from Sudan

**DOI:** 10.3390/microorganisms11061432

**Published:** 2023-05-29

**Authors:** Mohamed A. Hussain, Malik Suliman Mohamed, Hisham N. Altayb, Ahmed Osman Mohamed, Ahmed Ashour, Wadah Osman, Asmaa E. Sherif, Kholoud F. Ghazawi, Samar F. Miski, Sabrin R. M. Ibrahim, Gamal A. Mohamed, Ikhlas A. Sindi, Ahmad A. Alshamrani, Abdelaziz Elgaml

**Affiliations:** 1 Department of Pharmaceutical Microbiology, Faculty of Pharmacy, International University of Africa, Khartoum P.O. Box 2469, Sudan; ahmedkunna93@hotmail.com; 2 Department of Pharmaceutics, College of Pharmacy, Jouf University, Sakaka 72388, Saudi Arabia; msmustafa@ju.edu.sa; 3 Department of Pharmaceutics, Faculty of Pharmacy, University of Khartoum, Khartoum 11111, Sudan; 4 Department of Biochemistry, Faculty of Sciences, King Abdulaziz University, Jeddah 23589, Saudi Arabia; hdemmahom@kau.edu.sa; 5 Department of Pharmacognosy, Faculty of Pharmacy, Prince Sattam Bin Abdulaziz University, Alkharj 11942, Saudi Arabia; ahmedadelashour@yahoo.com (A.A.); w.osman@psau.edu.sa (W.O.); asmaasherif80@yahoo.com (A.E.S.); 6 Department of Pharmacognosy, Faculty of Pharmacy, Mansoura University, Mansoura 35516, Egypt; 7 Department of Pharmacognosy, Faculty of Pharmacy, University of Khartoum, Khartoum 11115, Sudan; 8 Clinical Pharmacy Department, College of Pharmacy, Umm Al-Qura University, Makkah 24382, Saudi Arabia; kfghazawi@uqu.edu.sa; 9 Department of Pharmacology and Toxicology, College of Pharmacy, Taibah University, Al-Madinah Al-Munawwarah 30078, Saudi Arabia; smiski@taibahu.edu.sa; 10 Department of Chemistry, Preparatory Year Program, Batterjee Medical College, Jeddah 21442, Saudi Arabia; sabrin.ibrahim@bmc.edu.sa; 11 Department of Pharmacognosy, Faculty of Pharmacy, Assiut University, Assiut 71526, Egypt; 12 Department of Natural Products and Alternative Medicine, Faculty of Pharmacy, King Abdulaziz University, Jeddah 21589, Saudi Arabia; gahussein@kau.edu.sa; 13 Department of Biology, Faculty of Science, King Abdulaziz University, Jeddah 21589, Saudi Arabia; easindi@kau.edu.sa; 14 Pharmaceutical Care Department, Ministry of National Guard–Health Affairs, Jeddah 22384, Saudi Arabia; shamraniah01@mngha.med.sa; 15 Microbiology and Immunology Department, Faculty of Pharmacy, Mansoura University, Mansoura 35516, Egypt; elgamel3a@mans.edu.eg; 16 Microbiology and Immunology Department, Faculty of Pharmacy, Horus University, New Damietta 34511, Egypt

**Keywords:** genomic analysis, multi-drug resistant, *Pseudomonas aeruginosa*, Sudan

## Abstract

*Pseudomonas aeruginosa (P. aeruginosa*) is known to be associated with resistance to practically all known antibiotics. This is a cross-sectional, descriptive, laboratory-based analytical study in which 200 *P. aeruginosa* clinical isolates were involved. The DNA of the most resistant isolate was extracted and its whole genome was sequenced, assembled, annotated, and announced, strain typing was ascribed, and it was subjected to comparative genomic analysis with two susceptible strains. The rate of resistance was 77.89%, 25.13%, 21.61%, 18.09%, 5.53%, and 4.52% for piperacillin, gentamicin, ciprofloxacin, ceftazidime, meropenem, and polymyxin B, respectively. Eighteen percent (36) of the tested isolates exhibited a MDR phenotype. The most MDR strain belonged to epidemic sequence type 235. Comparative genomic analysis of the MDR strain (GenBank: MVDK00000000) with two susceptible strains revealed that the core genes were shared by the three genomes but there were accessory genes that were strain-specific, and this MDR genome had a low CG% (64.6%) content. A prophage sequence and one plasmid were detected in the MDR genome, but amazingly, it contained no resistant genes for drugs with antipseudomonal activity and there was no resistant island. In addition, 67 resistant genes were detected, 19 of them were found only in the MDR genome and 48 genes were efflux pumps, and a novel deleterious point mutation (D87G) was detected in the *gyrA* gene. The novel deleterious mutation in the *gyrA* gene (D87G) is a known position behind quinolone resistance. Our findings emphasize the importance of adoption of infection control strategies to prevent dissemination of MDR isolates.

## 1. Introduction

*Pseudomonas aeruginosa (P. aeruginosa*) is an aerobic, non-spore forming, Gram negative rods gamma proteobacterium [1]. The genome size of *P. aeruginosa* can reach 7.3 Mb, which contains core genes plus variable accessory genes [2]. *P. aeruginosa* is frequently associated with a wide range of acute and chronic infections, particularly infections of chronic wounds, such as pressure ulcers, diabetic ulcers, and venous ulcers, in addition to pneumonia in cystic fibrosis and cancer patients taking chemotherapy [3,4]. *P. aeruginosa* causes hospital-acquired infections and, to a lesser extent, community-acquired infections as a result of its numerous virulence factors and resistance to many antimicrobials and antiseptics aided by its ubiquitous nature and ability to survive in low-nutrient environments [3,4,5]. The Center for Disease Control and Prevention (CDC) has announced the threat of multidrug-resistant (MDR) Gram negative bacteria [6]. A serious problem is the emergence of MDR-*P. aeruginosa* strains that are resistant to almost all known antimicrobial agents [3]. This bacterium possesses many genes that are responsible for intrinsic resistance to different classes of antimicrobial agents, along with the ability to acquire new genes [7].

The resistance to β-lactam antibiotics can be attained by the production of β-lactamase enzymes [8]. The most commonly acquired β-lactamases found among *P. aeruginosa* isolates are penicillinases belonging to the molecular class A serine β-lactamases (PSE, CARB, and TEM families) [9,10]. Extended-spectrum β-lactamases from the class D OXA-type enzymes have also been encountered within *P. aeruginosa* [11,12].

Acquired resistance to aminoglycosides basically involves enzymatic inactivation of the drug molecule via phosphoryltransferase, acetyltransferase, nucleotidyltransferase, or methylation of the *16S rRNA* [13,14,15]. The ability of *P. aeruginosa* to carry the genes for multiple aminoglycoside-inactivating enzymes provides individual strains with the potential to develop resistance to all aminoglycosides [13,14,15].

*P. aeruginosa* also can acquire resistance by an efflux mechanism and a reduction in drug accumulation that can be achieved through active expulsion by membrane-associated pumps [16]. The multidrug efflux pump with a broad specificity function might act synergistically with the outer membrane barrier to provide multi- or all-drug resistance [17,18]. *P. aeruginosa* genome analysis has revealed the presence of all five superfamilies of efflux systems, with the largest number from the RND family [19]. 

Resistance due to changes in the antibiotic target sites, such as penicillin-binding proteins (PBP), ribosomes, or increasing the activity of degrading enzymes, might happen due to mutations of the genes encoding these targets and enzymes [20,21]. Mutations that precipitate resistance to quinolones via reduced affinity of DNA gyrase to fluoroquinolone might occur through mutations in the *gyrA* gene, which encodes the A subunit of the target enzyme (DNA gyrase) [22,23,24,25]. Amino acid substitutions reported so far in *gyrA* gene, such as Ser-83-Leu and Asp-87-Asn, may play a vital role in quinolone resistance acquisition [24,26].

Data regarding MDR in Sudan is frightening; several studies have reported a spike in the incidence of MDR Gram negative bacteria in Sudan. There is, however, a paucity of data focused on the molecular basis behind *P. aeruginosa* resistance. Therefore, the aim of the present study was to identify MDR-*P. aeruginosa* isolates collected from different hospitals in Sudan and to typify and determine their genomic profiles.

## 2. Materials and Methods

This is a cross-sectional, laboratory-based, analytical study carried out during a period of two years (October 2014 to October 2016) that resulted in the collection of 385 primarily identified *P. aeruginosa* clinical isolates from different Sudanese hospitals in different states (1. Khartoum state: Soba University Teaching Hospital, Military Hospital, Gaffer Ibn Aoof Pediatric Hospital and Police Hospital. 2. Gezera state: Gizera University Central Laboratory. 3. Sinnar state: Insurance Health Center and Al-Suki Hospital. 4. Red Sea: Al-sharg National College. 5. North Darfur state: Insurance Health Center. 6. Blue Nile state: Al-damazin Teaching Hospital. 7. Al-gadarif state: Alg-adarif Teaching Hospital. 8. North Kordofan state: Alobaied Teaching Hospital. 9. River Nile state: MC Nimer Teaching Hospital).

### 2.1. Bacterial Identification

The collected samples were sub-cultured in nutrient agar (Himedia, Mumbai, India) for further phenotypic and genotypic tests. Isolates that showed the typical colonial morphology of *P. aeruginosa* (round, viscous, and pigmented colony) were checked for Gram staining. Gram negative isolates were sub-cultured in cetrimide agar (Rapid lab, Colchester, UK), and the isolates that showed growth were further confirmed by oxidase (Bioanalyse, Ankara, Turkey), catalase (Bells, Burton-on-Trent, UK), and citrate tests (Himedia, India). Several colonies from each isolate showing multiple drug resistance were removed and inoculated into an Eppendorf tube containing peptone water broth (Himedia India) with 20% of glycerol as a preservative, and were stored at −70 °C.

### 2.2. DNA Extraction

The genomic DNA of *P. aeruginosa* isolates were extracted using a QIAamp DNA minikit (Qiagen, Hilden, Germany). The quality and approximate quantity of the extracted DNA were performed using 2% agarose gel electrophoresis and a Nanodrop spectrophotometer (NanoDrop Technologies, Inc. Wilmington, DE, USA).

### 2.3. PCR

For molecular confirmation of the isolates, the *16S rRNA* gene was amplified using a thermocycler (SensoQuest GmbH, S/N 1320300144, Model LabCycler 48, SensoQuest GmbH D-37085, Hannah-Vogt-Str. 1, Goettingen, Germany). The following primer pairs, 27F 5′-AGAGTTTGATCCTGGCTCAG-3′ 149R 5′-CTACGGCTACCTTGTTACGA-3′, were used and prepared as instructed by the manufacturer [27]. The temperature/time adopted was an initial denaturation step at 94 °C for 5 min, followed by 35 cycles of denaturation at 94 °C for 1 min, annealing at 58 °C for 1 min, followed by a step of elongation at 72 °C for 1 min, and a final elongation at 72 °C for 5 min.

### 2.4. 16S rRNA Gene Sequencing

DNA sequencing was performed for the amplified 16S rRNA gene using the Sanger sequencing method (Macrogen, Seoul, Republic of Korea). The sequences were analyzed with the aid of BLAST and the intact sequences were deposited to NCBI to gain accession numbers. 

### 2.5. Antimicrobial Susceptibility Testing (AST)

Antimicrobial susceptibility testing was performed using the disc diffusion technique. The inoculum size was matched against 0.5 McFarland standards [28]. *P. aeruginosa* ATCC 27853 was used as a reference strain. Antibiotic discs used were gentamicin (10 µg), ceftazidime (30 µg), ciprofloxacin (5 µg), meropenem (10 µg), piperacillin (100 µg), and polymyxin B (300 units). After an incubation period, the inhibition zone diameters were measured and interpreted according to the clinical and laboratory standards institute (CLSI, 2015) guidelines. Isolates that showed an inhibition zone diameter ≤14 mm for ceftazidime, ≤15 mm for ciprofloxacin, ≤12 mm for gentamicin, ≤13 mm for meropenem, ≤17 mm for piperacillin, and ≤11 mm for polymyxin B were considered as resistant [28]. “MDR isolate was defined as acquired non-susceptibility to at least one agent in three or more antimicrobial categories” as per Magiorakos et al.’s recommendation [29]. Further, the multiple antibiotic resistance (MAR) index was determined for each isolate using the formula described previously: “MAR = a/b, where a represents the number of antibiotics to which the tested isolate depicted resistance, and b represents the total number of antibiotics to which the test isolate has been evaluated for susceptibility” [30].

### 2.6. Sequences Cleaning and Alignment

Finch TV (Geospiza, Seattle, WA, USA) was used to visualize the 16S ribosomal RNA gene sequence chromatogram and its quality and to confirm that all confusing sites are correctly called and determined [31]. Nucleotide BLAST (http://blast.ncbi.nlm.nih.gov/Blast.cgi) accessed on 3 February 2016, was used to search for sequence similarity of the obtained nucleotide sequences of the *16S rRNA* gene [32]. BioEdit 7.2 software was used to carry out multiple sequence alignment with highly similar sequences retrieved from NCBI [33]. Phylogenetic analysis was conducted using the PATRIC-BV-BRC server (https://www.bv-brc.org/) accessed on 12 February 2016; initially, the most similar and closest genomes were identified by Similar Genome Finder, and then the Bacterial Genome Tree was used to build the phylogenetic tree.

### 2.7. Whole-Genome Sequencing (WGS)

Whole-genome sequencing of the most MDR isolate was conducted by Macrogen Company (Seoul, Republic of Korea) using the Illumina Hiseq 2500 as the sequencing platform with a 101-bp read length for the paired-end read. The sequence data were filtered with a Phred score of >20. The genome was deposited to NCBI to obtain an accession number. 

### 2.8. Genome Assembly, Annotation, and Typing

FastQC program (https://www.bioinformatics.babraham.ac.uk/projects/fastqc/) accessed on 15 April 2016, was used for quality control of raw sequence data [34], while the adaptor sequences were trimmed from the edges of the reads using trimmomatic software version 0.32 [35]. The genome sequencing reads were de novo assembled by SeqMan NGen version 13.0.0 https://www.dnastar.com/software/genomics/ accessed on 2 May 2016, to convert them into contigs [36]. The NCBI Prokaryotic Genome Annotation Pipeline was used for automated genome annotation [37]. The sequence type of the selected isolate for WGS was carried out using multi-locus sequence typing and the results were compared with data from the MLST database (https://pubmlst.org/) accessed on 2 May 2016 [38].

### 2.9. Comparative Genomic

The whole genome of the selected MDR- *P. aeruginosa* was compared with database reference genomes from the genomic databases (*P. aeruginosa* strain POA1 (accession number: NC_002516.2) and *P. aeruginosa* strain VRFPA07 (accession number: AZBO00000000), which were reported to be susceptible to all commonly used drugs) [39]. Both genomes were retrieved from NCBI genomic database. The similarities/differences and content of genes across the genomes, percent of C and G content, and the number of non-coding genes, in addition to the distribution of coding regions across the genomes, were retrieved from the annotation of genomes of interest from NCBI. Mauve software version 2.3 was used to compare the three genomes in order to determine the core and accessory genes that are specific for each genome [40]. Progressive mauve software version 2.4.0 was used for contigs reordering against the reference genome to facilitate the visual comparison [41]. For the detection of a resistance island, PAIDB software v2.0 was used [42]. RAST server 2.0 was used for annotation of the genomic island present only in the MDR genome shown in this study [43]. Plasmid finder was used for plasmid sequence detection from the entire genome [44] and from the raw data using the plasmidSPAdes toolv3.15.4. PHASTER software was used for the detection of prophage sequences from the complete genome sequence [45]. Variant calling and annotation were performed using the Galaxy platform [46]. 

### 2.10. Antibiotic Resistance Genes Detection

Resistant Gene Identifier (RGI) (https://card.mcmaster.ca/analyze/rgi) accessed on 5 May 2016, was used for the detection of antibiotic resistance determinants in the assembled contigs [47]. 

### 2.11. Analysis of Novel Mutations in the Antibiotic-Resistant Genes

SNPs that reside within antimicrobial-resistant genes were checked for their novelty using nucleotide BLAST (http://blast.ncbi.nlm.nih.gov/Blast.cgi) accessed on 3 February 2016, [32]. Novel deleterious SNPs were further studied to forecast the influence of the point mutation on protein structure and stability using I-Mutant2.0 and Project HOPE web server, respectively [48,49]. Phyre2 (http://www.sbg.bio.ic.ac.uk/phyre2/) accessed on 1 May 2016, was used to predict the 3-dimensional structure of some important amino acid sequences [50]. Chimera version 1.9 software (https://www.cgl.ucsf.edu/chimera/) accessed on 1 May 2016, was used for visualization and prediction of the tertiary model and analysis of the molecular structures of the protein [51]. 

### 2.12. Statistical Analysis

Descriptive analysis for the data collected from experimental works were carried out using statistical analysis software (SAS 9.4), in addition to bioinformatics software for whole-genome and sequence analysis.

## 3. Results

### 3.1. Characteristics of the Collected Bacterial Isolates

From 385 pre-identified clinical isolates, only 200 isolates were confirmed as *P. aeruginosa*. Eighteen percent of the tested isolates exhibited a multidrug resistance phenotype. Additionally, one-third of the isolates showed a MAR index greater than 0.2 (Supplementary Table S4). Table 1 shows the results of antimicrobial susceptibility testing for six different antimicrobial agents with antipseudomonal activity, while Table 2 shows the pattern of resistance of the isolates for more than one tested antimicrobial simultaneously. 

Among piperacillin-resistant isolates, 84.3%, 71.6%, 65%, and 62.2% were susceptible to meropenem, ceftazidime, gentamicin, and ciprofloxacin, respectively, while only 54.5, 36.4, 36.4, and 0.00% were susceptible to ceftazidime, ciprofloxacin, gentamicin, and piperacillin, respectively, among meropenem-resistant isolates. In addition, 86.1% and 2.8% were found to be susceptible to meropenem and piperacillin, respectively, among ceftazidime-resistant isolates. Table 2 showed the susceptibility and resistant rates for different isolates. Further details of the co-resistance data are provided in Supplementary Table S5.

### 3.2. Amplified 16S rRNA 

**Figure 1:**
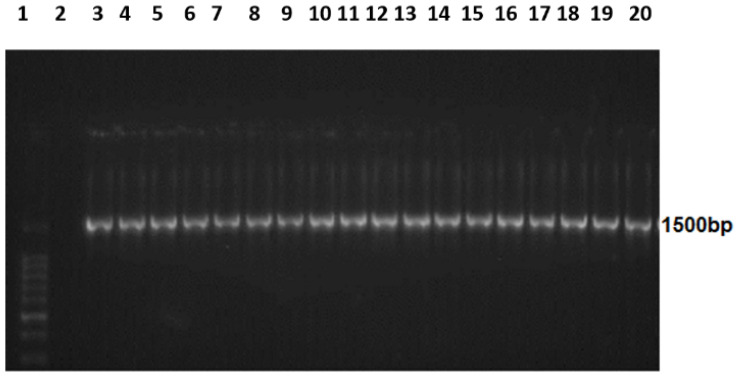
Amplified *16s rRNA* genes on 2% agarose gel electrophoresis. Lane 1 DNA ladder: MW 100–1500 bp. Lane 2 negative control. Lane 3–20 16 s ribosomal RNA genes (1500 bp) of the 18 isolates.

The 16S ribosomal RNA (1500 bp), which can be used for molecular characterization and to assess the evolutionary relationship between bacteria, was amplified using PCR and visualized using agarose gel electrophoresis (Figure 1). 

DNA sequencing was performed for *16S rRNA* genes and the successful sequences were deposited into the NCBI database. Supplementary Table S1 shows the accession numbers of 17 isolates. 

### 3.3. Comparative Genomic Results

The genome sequence was deposited to NCBI with accession number (MVDK00000000). The genome sequence of the selected MDR isolate was attributed to the sequence type (ST) 235 using multi-locus sequence typing (MLST database) [38]. The compared genomes showed variation in the genome size, number of genes, pseudogenes, proteins, CG%, rRNA, tRNA, ncRNA, and antimicrobial resistance genes. Table 3 shows the results produced by different software, such as the NCBI annotation pipeline accessed on 3 May 2014, and antibiotics resistant gene identifier (https://card.mcmaster.ca/analyze/rgi.) accessed on 5 May 2016. 

**Figure 2:**
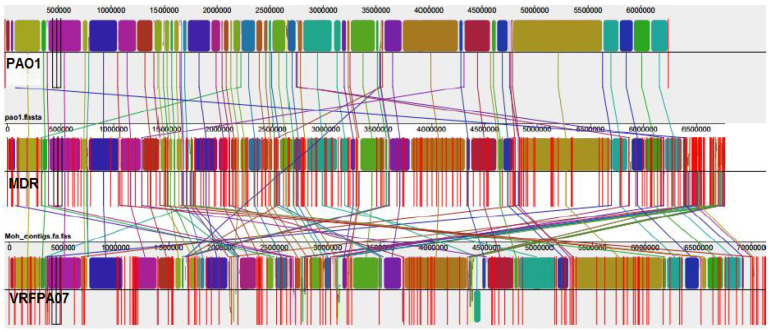
Alignment of *P. aeruginosa* PAO1 and MDR isolates and *P. aeruginosa* VRFPA07 genomes using MAUVE version 2.3. MDR and VRFPA07 genome sequences were rearranged to facilitate visual comparison prior to alignment. Homologous regions represented by the identically colored boxes are known as locally collinear blocks (LCBs). The inverted sequence of VRFPA07 relative to the other genomes is shown as green blocks below the horizontal line. The vertical lines linking the LCBs point among homologous regions of the three genomes. Numbers above the charts point to nucleotide positions within the corresponding genomes.

Mauve version 2.4.0 software analysis (Figure 2) revealed that there were variations in the genome sizes and that the MDR genome size diversity might have been caused by accessory DNA elements located in 40 regions that are scattered around the genome. Five of these regions were specific to the MDR genome. The conserved core component of the three genomes is largely collinear and exhibits slight intra-species diversity, which suggests that the *P. aeruginosa* genome has numerous strain-specific regions interspersed in a well conserved backbone.

By using resistant gene identifier web portal 6.0.1, 67 resistant genes have been detected in the MDR genome; 19 of them were non-efflux pump, while 48 of them were efflux pump resistant genes. Nineteen resistant genes (*aadA6*, *APH(3′)-V1*, *PDC-2*, *VEB-9*, *PmrA*, *PmrB*, *Mutant gyrA*, *cysB*, *alaS*, *ileS*, *MexS*, *mdtB*, *mdtC*, *nalC*, *nalD*, *NfxB*, *FloR*, *PmrA*, and *TetG*) were found only in the MDR genome. Table 4 shows the non-efflux pump resistant genes found in the three genomes of interest, while Table 5 shows ORFs encoding putative drug transporters found in the three genomes.

Phylogenomic analysis of our MDR strain and the closest strains obtained from the PATRIC server revealed that our strain is closer to the virulent strain *P. aeruginosa* VRFPA07, as shown in Supplementary Figure S1.

### 3.4. Detected Variants across Genomes

The whole genome sequencing and comparative genomics revealed the presence of numerous SNPs, insertions–deletions (indels), and multi-nucleotide polymorphisms (MNPs) in the entire MDR genome. This study revealed 49,551 substitutions in the entire MDR genome in comparison with PAO1. From the total substitutions, there were 45,017 SNPs, 254 insertions, 263 deletions, 3193 complex, and 931 MNPs. In addition, 42,653 of the total substitutions were found in the coding region and 6898 SNPs were in the noncoding region (intergenic region, 5′ UTR, and 3′ UTR). It has been found that 33,741 substitutions of the coding region were synonymous (resulted in the same amino acid), 8912 were missense (resulted in a different amino acid), and 33 were frameshift (the majority of ins/del resides in the noncoding regions). 

It is worth noting that a lot of missense variants, which could be responsible for resistance, have been detected in the antimicrobial resistance genes. Table 4 and Table 5 showed the results of mutant genes. 

In the substituted nucleotide column, the first letter is for PAO1 genome, second one for MDR genome, and the third one for VRFPA07 genome. 

**Figure 3:**
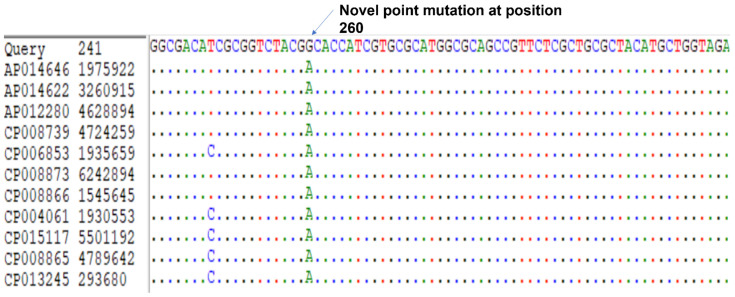
The novel point mutation at position 260 in the *gyrA* subunit.

**Figure 4:**
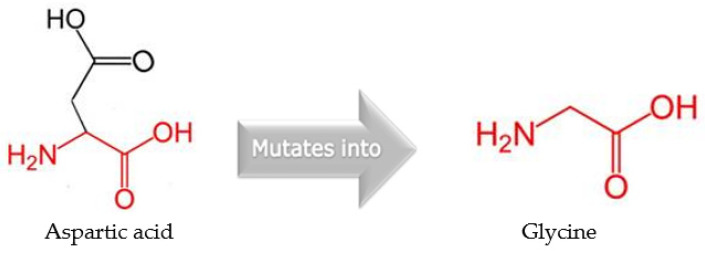
The schematic structure on the left is wild (original) type while on the right is the mutant amino acid at position 87. The backbone is the same for both amino acids, which is painted red. The side chain, unique for each amino acid, is painted black.

**Figure 5:**
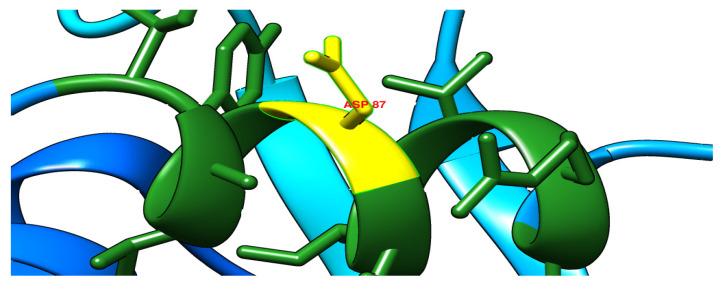
The three-dimensional structure of the wild type (aspartic acid) as it was predicted by Phyre2 software.

**Figure 6:**
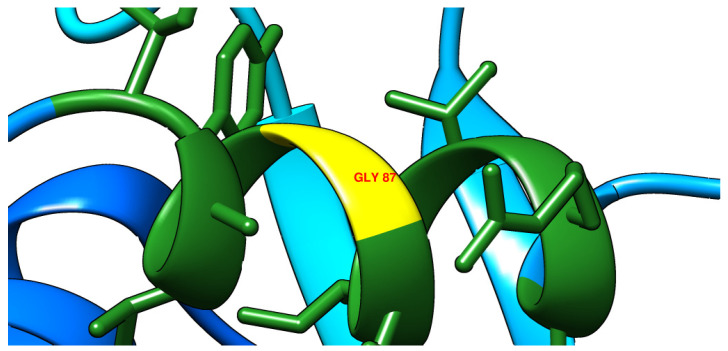
The three-dimensional structure of the mutant type (glycine) as it was predicted by Phyre2 software.

**Figure 7:**
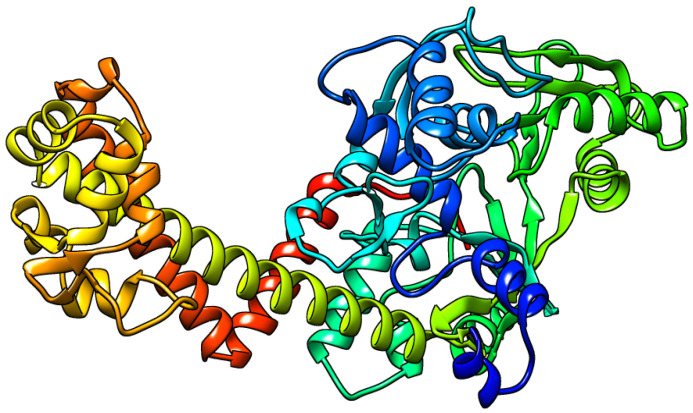
The three-dimensional structure of the complete protein of the mutant *gyrA* subunit as predicted by Phyre2.

A novel mutation in the *gyrA* gene from A to G at position 260 has been detected (Figure 3), and this led to an important amino acid substitution (D87G), which was predicted to affect the protein stability using I-Mutant2.0 software (Table 6) and protein structure using Project hope web server and Phyre2 software (Figure 4, Figure 5, Figure 6 and Figure 7). 

## 4. Discussions

The challenging pathogenic Gram-negative bacterium *P. aeruginosa* is known to withstand various environmental conditions and cause infections in almost all major body systems [52]. We collected 385 pre-identified *P. aeruginosa* clinical isolates from different Sudanese hospitals over a period of 2 years to study their antimicrobial sensitivity patterns and determine the genomic profile of the most resistant isolate. All isolates were re-examined and only 200 isolates were confirmed as *P. aeruginosa* using both phenotypic and genotypic techniques; this might be attributed to the fact that not all *16S rRNA* genes can be successfully amplified due to primers, PCR conditions used, or other reasons. The deficiency of laboratory facilities for rapid and ideal identification at the strain level, and sometimes, misidentification of *P. aeruginosa* have remarkable consequences for the patients regarding morbidity and cost-effectiveness use of antibiotics [53,54]. A high rate of resistance to front-line agents has been detected among the 200 tested isolates according to the CLSI susceptible breakpoint (Table 1). The respective rate of susceptibility to major antimicrobial agents was as follows: ceftazidime (79.4%), ciprofloxacin (73.4%), and gentamicin (71.9%). Furthermore, 36 (18%) of the tested isolates exhibited an MDR phenotype, which could be regarded as an alarming rate of resistance for this widely distributed bacterium. Among the antipseudomonal agents tested, meropenem and polymyxin B showed 90.96% and 94.47% activity against the tested isolates, respectively, and this might be attributed to the restricted use of these drugs in the study area. It is known that polymyxin B is associated with serious side effects and toxicity, so its systemic use is restricted to infection refractory to other antipseudomonal drugs [55]. Likewise, meropenem is too expensive, which makes it unaffordable by the majority of the patients in less developed countries. 

We thought that over-use of antibiotics might accelerate the rate of resistance; however, we found that piperacillin, which is not yet registered in Sudan, showed the highest rate of resistance among all tested drugs (166, 77.89%) (Table 1). This might be attributed to cross border transmission of resistant bacteria with travelers or goods. In addition, resistance can occur naturally or by misuse of drugs, and there are reports about the high prevalence of the *ESBL* gene in Sudan [56]. 

The low rate of ciprofloxacin susceptibility to gentamicin-resistant isolates detected in this study (Table 2) may be attributed to presence of *aac(6′)-Ib-cr*, which is an aminoglycoside acetyltransferase gene that can also inactivate ciprofloxacin [57]. It has been reported that both gentamicin-resistance and piperacillin-resistance were more common among fluoroquinolone resistant isolates, which might be due to the fact that the majority of fluoroquinolone-resistant isolates carry the *CTX-M* gene [58,59]. 

The MLST system, which is known to be highly discriminatory in the identification of bacteria, has been used in this study for the identification of *P. aeruginosa* at the strain level [60,61,62]. The identified isolate was ascribed to ST-235 according to the MLST database. This strain is widely distributed in the world and was reported in more than 17 countries [38]. 

We compared the genome of the MDR isolate with the reference genomes from the NCBI and *P. aeruginosa* database in order to explore the common features and mechanisms by which resistance occurs. The three genomes are remarkably similar, although there are slight variations in the genome sizes; the conserved core component of the three genomes is largely collinear and exhibits slight intra-species diversity, which suggests that the *P. aeruginosa* genome has numerous strain-specific regions interspersed in a well conserved backbone. The size diversity of the studied MDR genome is mainly caused by accessory DNA elements located in 40 regions that are scattered around the genome (Figure 2). It is interesting to note that five of these regions were detected in the current MDR genome only. The genome annotation and analysis predicted that the main genes in these five regions are hypothetical proteins, beside genes involved in metabolic and biological processes together with two genes for quaternary ammonium compound resistance and the arsenical pump driving ATPase. The analysis also revealed that the MDR genome contains no resistant island as it has not been detected by PAIDB v2.0 software [42], and it contains one plasmid but, surprisingly, does not carry any known resistance markers for antipseudomonal drugs. Nonetheless, it contains only one operon of the mercury resistant regulatory protein, which is known in other bacteria [63]. We believe that antibiotic resistant genes are interspersed with mobile genetic elements in the bacterial chromosome. 

It has been observed that the studied MDR genome has a low CG% (64.6) content when compared with other genomes of interest (Table 3). This could make the MDR strain more susceptible to horizontal gene transfer [64]. Moreover, the annotation of the sequenced MDR genome revealed the presence of a prophage sequence (phage_Pseudo_phiCTX_NC_003278(5) [45], and the annotation of the sequences surrounding the prophage sequence revealed the presence of genes responsible for metabolic and biological processes. 

We detected 19 antimicrobial resistant genes that were absent in the reference genomes. The identified AMRGs were 67, 62, and 59 putative ORFs in the MDR strain, VRFPA07, and PAO1 genomes, respectively (Supplementary Tables S2 and S3). It is worth noting that the MDR strain was resistant to meropenem, piperacillin, ceftazidime, gentamicin, and ciprofloxacin (Table 1). Resistance to β-lactam antibiotics may be mediated through the ESBL enzyme class D *OXA-50*, which can inactivate meropenem, piperacillin, and cephalosporin, *VEB-9*, which is class A ESBL and can inactivate cephalosporin, and the extended spectrum cephalosporinase *PDC-2*, which can inactivate carbapenem and cephalosporin [65]; we believe that these β-lactamases are behind the resistance detected in the studied MDR strain.

The ability of the MDR isolate to resist aminoglycosides could be through the presence of *APH(3′)-VI*, which is an aminoglycoside phosphotransferase that can inactivate a wide range of aminoglycosides, *APH(3)-IIb* aminoglycoside phosphotransferase, which can inactivate kanamycin and neomycin, and the aminoglycoside-adenyletransferase *aadA6*, which inactivates streptomycin and spectinomycin [47]. We detected polymyxin resistant determinants (*PmrA*, *PmrB*, *PmrC*, *PmrF*, *Arna*) in the three genomes (Supplementary Tables S2 and S3); however, the MDR strain was sensitive to polymyxin, which might explain why these genes were not expressed or that there is deficiency in some modulators that help in polymyxin resistance. 

Forty-eight of the ORFs detected were encoded by putative efflux pumps from the RND family (36 ORFs), MFS family (7 ORFs), MATE family (1 ORF), SMR family (1 ORF), and ABC superfamily (3 ORFs) (Supplementary Tables S2 and S3). It is known that efflux pumps are an important cause of antimicrobial resistance in bacteria and might partially explain the MDR of the isolate under study [18,66,67]. Nine of these efflux pumps (*nfxB*, *nalD*, *nalC*, *mexS*, *mdtC*, *mdtB*, *tetG*, *floR*, and *emeR*) are present in the study isolate and are absent from the other genomes of interest. This finding supports the report stated that RND is the family that is expressed abundantly in *P. aeruginosa* [17]. 

A point mutation in the MexR efflux pump at position T377A, which leads to substitution of Val126Gln, has been detected in the MDR isolate (Table 5). This substitution has been reported to be behind antibiotic resistance because it leads to the overexpression of MexAB-Oprm [68,69] and is negatively regulated by mexR [70]. Furthermore, we detected two missense mutations in mexD Ile982Val and Ser845Ala (Table 5). These mutations have been reported to impact the transport of numerous substrates such as β-lactams antibiotics as well as several cytoplasmic acting antimicrobials [71]. 

Quinolone resistance is mainly attributed to the nucleotide point mutation at position 87, a known position behind the quinolone resistance, of the gene that encodes the mutant *gyrA* [24]. The presence of target protein protection (*mfd* gene) also could contribute to quinolone resistance. The novel mutation detected in the *gyrA* gene in this study, A260G, has been found to convert aspartic acid to glycine (D87G) (Figure 3). This mutation has a profound impact at different levels on the binding affinity of ciprofloxacin to DNA gyrase, notably, the difference between the wild-type residue and the mutant one in terms of size, charge, and hydrophobicity-value; and consequently, changing the structural conformation and affecting the binding affinity between quinolone and DNA gyrase [49].

Despite the identified mutations, the whole genome sequence has not shown mutations in the other parts of the QRDR, such as *gyrB*, *parC*, and *parE*. This evidence, together with the previous report [72], powerfully supports the finding that the mutant *gyrA* (D87G) could be behind the quinolone resistance. 

## 5. Conclusions

From 385 pre-identified isolates, 200 were confirmed as *P. aeruginosa* and their *16S rRNA* gene sequences were deposited to the NCBI GenBank database. The tested isolates showed variable responses to antipseudomonal antibiotics and 18 percent exhibited an MDR phenotype. The most resistant MDR strain was selected and its whole genome sequence was obtained using the NGS technique. We detected 19 antimicrobial resistant genes and 48 efflux pump genes in the MDR genome. There were some mutations detected in some resistant genes, such as the novel mutation of the *gyrA* gene (A260G) and MexR efflux pump gene (T377A), that were predicted to affect the protein stability and expected to be behind the resistance. Future studies should find answers to the sensitivity of some strains to some antimicrobials with the presence of relative resistant genes in their genomes. However, it should be noted that only one genome of the MDR isolate was sequenced due to cost constraints. Characterization of DNA sequence variation in *P. aeruginosa* is needed to define strain-specific sequences, determine level of expression of efflux pump genes, and analyze the effect of mutations in noncoding regions of prokaryotic systems. This may promote our understanding of genetic determinants of resistance mechanisms and enhance the understanding of the exact mechanism behind antibiotics resistance. There are many hypothetical proteins that exist in the MDR genome, but their exact functions need to be assigned. 

## Figures and Tables

**Table 1:** Antimicrobial susceptibility testing (*n* = 200).

Antibiotic	Sensitive %	Intermediate %	Resistant %
CIP 5 µg	(148) 74	(10) 5.0	(42) 21
PB 300 IU	(189) 94.5	(2.0) 1.0	(9.0) 4.5
PRL 100 µg	(44) 22	(0.0) 0.00	(156) 78
MEM 10 µg	(184) 92	(5.0) 2.5	(11) 5.5
CN 10 µg	(144) 72	(6.0) 3.0	(50) 25
CAZ 30 µg	(159) 79.5	(5.0) 2.5	(36) 18

CIP = ciprofloxacin, PB = polymyxin B, PRL = piperacillin, MEM = meropenem, CN = gentamicin, CAZ = ceftazidime, S= sensitive, I = intermediate, R = resistant.

**Table 2:** Co-resistance of isolates among the tested antimicrobials.

Co-Resistance	Number/Percent
PRL-MEM	(11) 5.5%
PRL-CAZ	(35)17.5%
MEM-CAZ	(5.0) 2.5%
PRL-MEM-CAZ	(5.0) 2.5%
PRL-CIP	(40) 20%
PRL-CN	(47) 23.5%
PRL-PB	(8.0) 4.0%
CIP-CN	(37) 18.5%

CIP = ciprofloxacin, PB = polymyxin B, PRL = piperacillin, MEM = meropenem, CN = gentamicin, CAZ = ceftazidime, S= sensitive, I = intermediate, R = resistant.

**Table 3:** Comparative genomic analysis among three genomes (MDR isolate, VRFPA07, and PAO1).

Strain Features	MDR Isolate	Susceptible (VRFPA07)	Reference Genome (PAO1)
NCBI accession no.	MVDK00000000	AZBO00000000	NC_002516.2
Genome coverage	132	80	---
Genome size (bp)	6,764,168	7,177,216	6,264,404
Contigs (n)	240	140	---
G + C content (%)	64.6	65.90	66.6
Genes (n)	6557	6916	5697
Pseudogenes (n)	120	84	19
Proteins (n)	6373	6765	5572
rRNAs (n)	4 (5S,16S,23S)	9 (5S, 16S, 23S)	13 (5S, 16S, 23S)
tRNAs (n)	56	57	63
ncRNAs (n)	4	1	30

**Table 4:** Antimicrobial resistant genes containing missense variants.

Gene Name	Number of Missense	Substituted Nucleotide	Substituted Amino Acid	Novelty/Effect
*OXA-50*	2	A46G	Thr16Ala	Reported
		A74G	Gln25Arg	Reported
				
*mfd*	1	G1171C	Ala391Pro	Reported
				
*gyrA*	2	A260G	asp87gly	Novel/deleterious
		G83A	thr83Ile	Reported/deleterious
*parC*	1	C260T	Ser87Leu	Reported/deleterious
				
*APH(3′)-IIb*	1	C128A	Ala43Glu	Reported

**Table 5:** Missense variants in the drug transporters that are shared in the three genomes of interest.

Gene Name	Number of Missense	Substituted Nucleotide	Substituted Amino Acid
*MexB*	2	2870GAG	Gly957Asp
		3120GTG	Ala1040Glu
			
*MexC*	5	1147GAG	Pro383Ser
		988ACC	Ser330Ala
		929TCT	His310Arg
		227CTC	Arg76Gln
		92GAG	Ala31Val
			
*MexD*	2	2944CTC	Ile982Val
		2533ACC	Ser845Ala
			
*MexE*	3	5ATA	Glu2Val
		23CTC	Ser8Phe
		1103AGA	Gln368Arg
			
*MexJ*	1	940CGG	Ala314Pro
			
*MexH*	1	906CAA	Asp302Glu
			
*MexI*	1	234CAA	Ala78Glu
			
*MexM*	4	140TAA	Ile47Asn
		689TCC	Leu230Pro
		974ACC	Asp325Ala
		1139CTC	Ala380Val
			
*MexN*	3	278CGC	Thr93Ser
		2351AGG	Ser784Phe
		3067AGG	Thr1023Ala
			
*MexP*	2	1097CAA	Arg366Leu
		890GTT	Ala297Glu

*MexQ*	4	1967CTT	Arg656Lys
		1514ACTT	Gly505Asp
		1150CTT	Val38Ile
		880CAA	Ile294Val
*MexV*	3	673GTG	Ala225Ser
		686CGG	Ala229Gly
		962AGG	Gln321Arg
			
*MexW*	2	779GTG	Arg260Gln
		1532AGG	Gln511Arg
			
*OprJ*	2	800TCT	Gly267ARg
		205TCT	Met69Val
			
*OpmD*	2	335GAA	Ser112Asn
		805GAG	Gly269Ser
			
*OprN*	1	37TCC	Ser13Pro
			
*OpmE*	3	1072CTC	Ala358Thr
		1060AGG	Trp354Arg
		523ATT	Ser175Thr
			
*AmrA*	4	1072AGG	Trp358Arg
		991ACC	Leu331Val
		985TGG	Lys329Gln
		88CTC	Ala30Thr
			
*AmrB*	1	1627TCC	Thr543Ala
			
*TriA*	5	872ACC	Glu291Ala
		911TAT	Vla304Asp
		942CTC	Asp314Gln
		C956TC	Arg319Val
		76GAA	Gly26Ser
			
*TriC*	1	1019GAA	Arg340Gln

**Table 6:** I-Mutant2.0 software results concerning stability of the mutant *gyrA*.

Position	WT	NEW	pH	Temp	SVMs Prediction Effect	DDG Value Prediction
87	D	G	7.0	25	Decrease	–0.75 Kcal/mol

## Data Availability

The data used to support the findings of this study are available from the corresponding author upon request.

## Supplementary Materials

The following supporting information can be downloaded at: https://www.mdpi.com/article/10.3390/microorganisms11061432/s1, Table S1: Accession numbers of *16S rRNA* genes deposited to the GenBank database; Table S2: ORFs encoding putative drug transporters of the three genomes of interest; Table S3: ORFs putatively associated with resistance to antibiotics in the three genomes; Table S4: multiple antibiotic resistance index of *P. aeruginosa* isolates; Table S5: co-resistance of the *P. aeruginosa* against the tested antibiotics.; Figure S1: Phylogenetic analysis of our MDR strain (indicated by red circle) and the most closest strains obtained from PATRIC server.
