# Supplementary material for: Comparative Genomic Analysis of Multi-Drug Resistant Pseudomonas aeruginosa Sequence Type 235 Isolated from Sudan
Source: Microorganisms. 2023 May 29;11(6):1432. doi: 10.3390/microorganisms11061432 (PMC10303892; doi:10.3390/microorganisms11061432)
Supplement: Supplementary file 1 [file microorganisms-11-01432-s001.zip › microorganisms-2354004-supplementary.pdf]

microorganisms

Article

Supplementary Materials

Comparative Genomic Analysis of Multi-Drug Resistant *Pseudomonas aeruginosa* Sequence Type 235 Isolated from Sudan

Mohamed A. Hussain [1,*], Malik Suliman Mohamed [2,3], Hisham N. Altayb [4], Ahmed Osman Mohamed [1], Ahmed Ashour [5,6], Wadah Osman [5,7], Asmaa E. Sherif [5,6], Kholoud F. Ghazawi [8], Samar F. Miski [9], Sabrin R. M. Ibrahim [10,11], Gamal A. Mohamed [12], Ikhlas A. Sindi [13], Ahmad A. Alshamrani [14] and Abdelaziz Elgaml [15,16]

[1] Department of Pharmaceutical Microbiology, Faculty of Pharmacy, International University of Africa, Khartoum P.O. Box 2469, Sudan; ahmedkunna93@hotmail.com
[2] Department of Pharmaceutics, College of Pharmacy, Jouf University, Sakaka 72388, Saudi Arabia; msmustafa@ju.edu.sa
[3] Department of Pharmaceutics, Faculty of Pharmacy, University of Khartoum, Khartoum 11111, Sudan
[4] Department of Biochemistry, Faculty of Sciences, King Abdulaziz University, Jeddah 23589, Saudi Arabia; hdemmahom@kau.edu.sa
[5] Department of Pharmacognosy, Faculty of Pharmacy, Prince Sattam Bin Abdulaziz University, Alkharj 11942, Saudi Arabia; ahmedadelashour@yahoo.com (A.A.); w.osman@psau.edu.sa (W.O.) asmaasherif80@yahoo.com (A.E.S.).
[6] Department of Pharmacognosy, Faculty of Pharmacy, Mansoura University, Mansoura 35516, Egypt
[7] Department of Pharmacognosy, Faculty of Pharmacy, University of Khartoum, Khartoum 11115, Sudan
[8] Clinical Pharmacy Department, College of Pharmacy, Umm Al-Qura University, Makkah 24382, Saudi Arabia; kfghazawi@uqu.edu.sa
[9] Department of Pharmacology and Toxicology, College of Pharmacy, Taibah University, Al-Madinah Al-Munawwarah 30078, Saudi Arabia; smiski@taibahu.edu.sa
[10] Department of Chemistry, Preparatory Year Program, Batterjee Medical College, Jeddah 21442, Saudi Arabia; sabrin.ibrahim@bmc.edu.sa
[11] Department of Pharmacognosy, Faculty of Pharmacy, Assiut University, Assiut 71526, Egypt
[12] Department of Natural Products and Alternative Medicine, Faculty of Pharmacy, King Abdulaziz University, Jeddah 21589, Saudi Arabia; gahussein@kau.edu.sa
[13] Department of Biology, Faculty of Science, King Abdulaziz University, Jeddah 21589, Saudi Arabia; easindi@kau.edu.sa
[14] Pharmaceutical Care Department, Ministry of National Guard–Health Affairs, Jeddah 22384, Saudi Arabia; shamraniah01@mngha.med.sa
[15] Microbiology and Immunology Department, Faculty of Pharmacy, Mansoura University, Mansoura 35516, Egypt; elgamel3a@mans.edu.eg
[16] Microbiology and Immunology Department, Faculty of Pharmacy, Horus University, New Damietta 34511, Egypt

* Correspondence: mkasamber@gmail.com

microorganisms MDPI

Table S1: Accession numbers of 16S rRNA genes deposited to GenBank database.

**Isolate number**	**Isolate**	**Accession number**
Isolate-1	8	KY549636
Isolate-2	11	KY549637
Isolate-3	38	KY549638
Isolate-4	44	KY549639
Isolate-5	45	KY549640
Isolate-6	62	KY549641
Isolate-7	64	KY549642
Isolate-8	81	KY549643
Isolate-9	82	KY549644
Isolate-10	92	KY549645
Isolate-11	100	KY549646
Isolate-12	101	KY549647
Isolate-13	102	KY549648
Isolate-14	103	KY549649
Isolate-15	144	KY549650
Isolate-16	146	KY549651
Isolate-17	158	KY549652

Table S2: ORFs encoding putative drug transporters of the three genomes of interest.

Transporter gene family	Gene name	Gene mediates resistance to	MDR isolate	VRFPA07	PAO1
**RND family**	adeL	Tetracycline, fluoroquinolone	-	+	+
	AmrA	aminoglycosides	+	+	+
	AmrB	aminoglycosides	+	+	+
	MexA	Peptide, sulfo, fluor, macro, tetr, carp, cephalo, phenicol monobactam, penam.	+	+	+
	MexB	Peptide, sulf, fluor, macro, tetr, carpa, cephalo, phenicol, monobactam, penam, penem, chephmycin	+	+	+
	MexC	fluor, macro, tetr, carpa, cephalo, phenicol, aminogl, penam,	+	+	+
	MexD	fluor, macro, tetr, carpa, cephalo, phenicol, aminogl, penam,	+	+	+
	MexE	Fluoro, pheniccol, diaminpyrimidine	+	+	+
	MexF	Fluoro, pheniccol, diaminpyrimidine	+	+	+
	MexG	Tetr, fluoro, acridine	+	+	+
	MexH	Tetr, fluoro, acridine	+	+	+
	MexI	Tetr, fluoro, acridine	+	+	+
	MexJ	Macro, tetr, triclosan	+	+	+
	MexK	Macro, tetr, triclosan	+	+	+
	MexL	Macro, tetr, triclosan	+	+	+
	MexM	phenicol	+	+	+
	MexN	phenicol	+	+	+
	MexP	Macro, carpab, acridine, tetr, diaminopyrimidine	+	+	+
	MexQ	Macro, carpab, acridine, tetr, diaminopyrimidine	+	+	+
	MexS	Phenicol, fluoro, diaminopyriimidine	+	-	-
	MexV	Macro, phenicol, acridine, tetr, fluoro	+	+	+
	MexW	Macro, phenicol, acridine, tetr, fluoro	+	+	+
	MuxA	Macro, tetr, monoba, aminocoumarin	-	+	+

	MuxB	Macro, tetr, monoba, aminocoumarin	-	+	+
	MuxC	Macro, tetr, monoba, aminocoumarin	-	+	+
	OpmB	Macro, tetr, monoba, aminocoumarin	-	+	+
	OpmD	Tetr, flouro, acridine	+	+	+
	OpmE	Phenicol, acridine, macro, tetr, carbap	+	+	+
	OpmH	triclosan	+	+	+
	OprJ	Aminog, tetr, phenicol, macro, fluoro, cephalo. Penam, aminocuom	+	+	+
	OprM	Sulf, fluoro, aminog, macro, carbap, tetr, monob, cephalo, phenicol, pepti, aminocoum, cephamycin	+	+	+
	OprN	Fluoro, phenicol, diaminopyrimidine	+	+	+
	CpxR	Sulf, fluoro, aminog, macro, carbap, tetr, monob, cephalo, phenicol, pepti, aminocoum, cephamycin	-	-	+
	TriA	triclosan	+	+	+
	TriB	triclosan	+	+	+
	TriC	triclosan	+	+	+
	mdtB	aminocoumarin	+	-	-
	mdtC	aminocoumarin	+	-	-
	nalC	Pept, sulf, penam, macro, fluoro, carpab, tetr, phenicol, cephalosp, penem, monob, aminocou	+	-	-
	nalD	Sulf, fluoro, aminog, macro, carbap, tetr, monob, cephalo, phenicol, pepti, aminocoum, cephamycin, penam diaminprimidine	+	-	-
	NfxB	Tetr, phenicol, fluoro, macro, penam, cephalosp, aminocoum, diaminopyrimidine	+	-	-

**MFS family**	FarB	Mediate resistance to antibacterial-free fatty A	+	+	+
	RosB	peptide	+	+	+
	EvgS	Tetr, macro, fluoro	+	+	+
	FloR	phenicol	+	-	-
	PmrA	fluoro	+	-	-
	TetG	tetr	+	-	-
**ABC family**	MsbA	Nitroimidazole	+	+	+
	macA	macrolide	+	-	+
	macB		+	+	+
**SMR family**	EmrE	Small multidrug resistance	+	-	+
**MATE family**	PmpM	Multidrug & toxic copounds	-	+	+

microorganisms MDPI

Table S3: ORFs putatively associated with resistance to antibiotics in the three genomes.

Antibiotic class	Gene name	Gene mediates resistance to	MDR isolate	VRFPA 07	PAO1
**Aminoglycosides**	aadA6	Aminoglycoside adenylyltransferase Mediate resistance to streptomycin & spectinomycin	+	-	-
	APH(3′)-V1	Aminoglycoside phosphotransferase	+	-	-
	APH(3′)-IIb	Aminoglycoside phosphotransferase Mediate resistance to kanamycin A&B, Neomycin B&C	+	+	+
**B-Lactam antibiotics**	PDC-1	Cephalosporin, monobactam, carbapenem,	-	-	+
	PDC-2	B-Lactamase, mediate resistance to carbapenem, cephalosporin & monobactam	+	-	-
	PDC-7	Cephalosporin, monobactam, carbapenem	-	+	-
	VEB-9	B-Lactamase, mediate resistance to cephalosporin &monobactam	+	-	-
	OXA-50	B-Lactamase mediate resistance to meropenem, ampicillin, piperacillin, cephalosporin & ticarcillin	+	-	+
**Polymyxins**	PmrA	Mediate resistance to polymyxins	+	-	-
	PmrB	Mediate resistance to polymyxins	+	-	-
	PmrC	Mediate resistance to polymyxins	+	+	+
	PmrF	Mediate resistance to polymyxins	+	+	+
	Arna	Mediate resistance to polymyxins	+	+	+
**Chloramphenicol**	CatB7	Chloramphenicol-acetyltransferase	+	+	+
**Fluoroquinolone**	Mfd	Target protection protein	+	+	+
	Mutant gyrA	Alteration of binding site	+	-	-

**Fosfomycin**	fosA	Fosfomycin thio-transferase	+	+	+
**Aminocoumarin**	cysB	Aminocoumarin & novobiocin resistance gene	+	-	-
	alaS	Aminocoumarin target alteration	+	-	-
**Muprocin**	ileS	Muprocin resistance gene	+	-	-
**Peptide antibiotic**	mprF	Peptide antibiotic	+	+	+

Table S4: multiple antibiotic resistance index of *P. aeruginosa* isolates.

MAR index	Frequency	Percentage
0.0	39	19.5 %
0.2	93	46.7 %
0.3	21	10.6 %
0.5	23	11.6 %
0.7	20	10.1 %
0.8	4	2 %
**Total**	**200**	**100 %**

Table S5: co-resistance of the *P. aeruginosa* against the tested antibiotics.

Co-resistance	Frequency	Percent
**CIP-PRL**	4	2.0
**PB-PRL**	1	0.5
**PRL-MEM**	3	1.5
**CIP-CN**	2	1.0
**PB-CN**	1	0.5
**PRL-CN**	2	1.0
**CIP-PRL-CN**	12	6.0
**PB-PRL-CN**	5	2.5
**PRL-MEM-CN**	1	0.5
**CIP-PRL-MEM-CN**	2	1.0
**PRL-CAZ**	8	4.0
**PB-PRL-CAZ**	1	0.5
**CIP-PRL-MEM-CAZ**	1	0.5
**PRL-CN-CAZ**	4	2.0
**CIP-PRL-CN-CAZ**	17	8.5
**CIP-PRL-MEM-CN-CAZ**	4	2.0
**Total number of co-resistance**	68	34

CIP = Ciprofloxacin, PB = Polymyxin B, PRL = Piperacillin, MEM = Meropenem, CN = Gentamicin, CAZ = Ceftazidime, S= sensitive, I = intermediate, R = resistant.

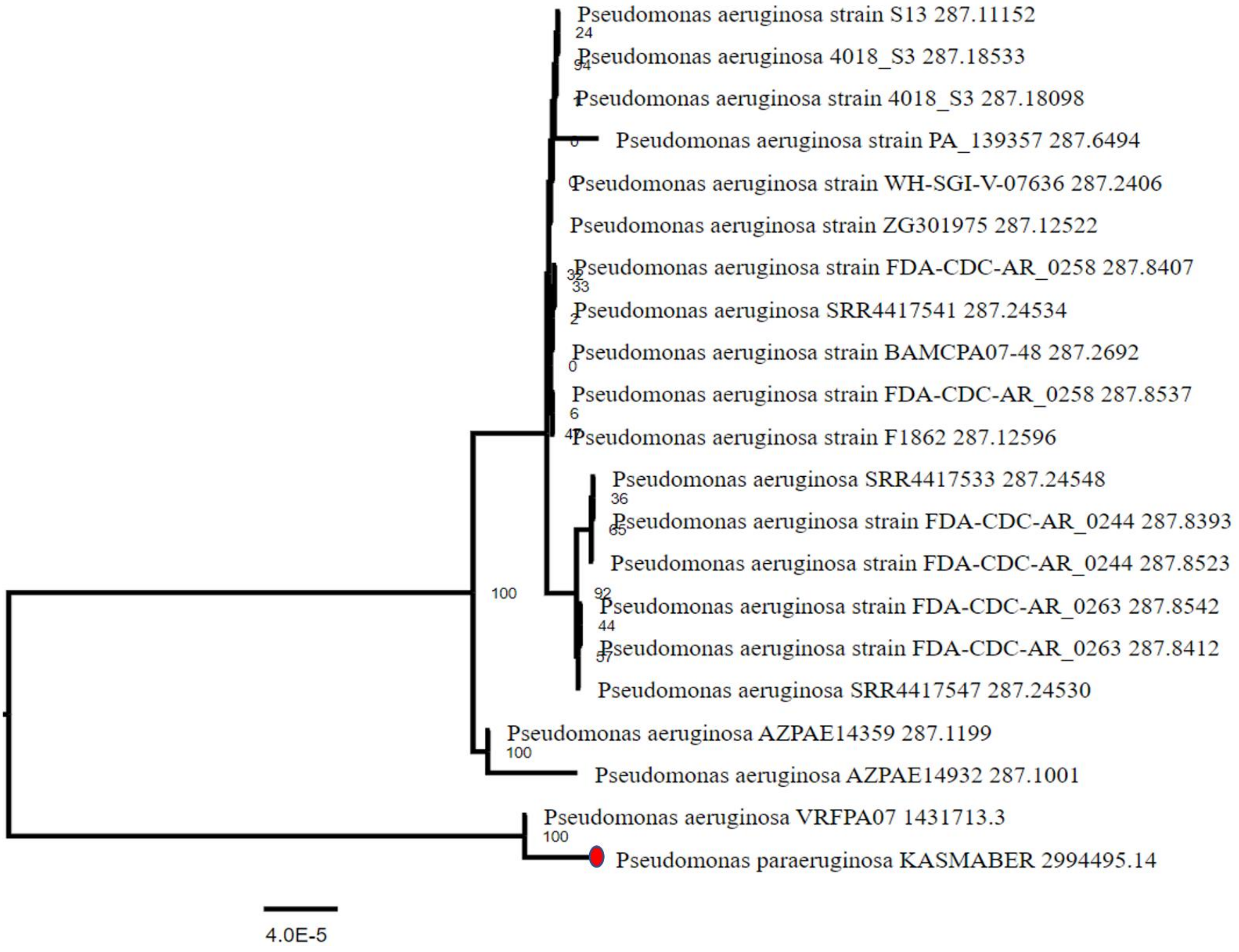

Figure S1. Phylogenetic analysis of our MDR strain (indicated by red circle) and the most closest strains obtained from PATRIC server.
